# Microarray dataset of after-ripening induced mRNA oxidation in wheat seeds

**DOI:** 10.1016/j.dib.2018.10.036

**Published:** 2018-10-16

**Authors:** Feng Gao, Mark C. Jordan, Belay T. Ayele

**Affiliations:** a Department of Plant Science, University of Manitoba, 222 Agriculture Building, Winnipeg, Manitoba, Canada R3T 2N2; b Morden Research and Development Centre, Agriculture and Agri-Food Canada, Morden, Manitoba, Canada R6M 1Y5

**Keywords:** After-ripening, Dormancy, Microarray, mRNA oxidation, Seed, Wheat

## Abstract

The dataset presented here profiles oxidative modification of mRNAs in wheat seeds in response to after-ripening, a treatment that releases seeds from the state of dormancy. The level of dormancy in wheat seeds is closely associated with preharvest sprouting, defined as the germination of seeds while they are on the mother plant, which negatively affects wheat yield and quality. Understanding the molecular mechanisms involved in the control of seed dormancy is critical for improving the tolerance of wheat seeds to preharvest sprouting. The dataset were generated using oxidized mRNA samples derived from three independent biological replicates of dormant and after-ripened (non-dormant) wheat seeds and a microarray based experimental procedures that involved the use of Affymetrix GeneChip Wheat Genome Array. The raw and normalized data are available in NCBI׳s Gene Expression Ominbus (GEO) database with accession number GSE41949, and a related research article has been published in Plant Biotechnology Journal (Gao et al., 2013).

**Specifications table**

**Table:** 

Subject area	Biology
More specific subject area	Oxidative modification of mRNAs
Type of data	Data Table in Excel and CEL files
How data was acquired	Affymetrix GeneChip Wheat Genome Array
Data format	Raw and normalized data
Experimental factors	Dormant seeds vs. after-ripened (non-dormant) seeds
Experimental features	Isolation of oxidized mRNAs, large scale analysis of oxidative modification of seed stored mRNAs
Data source location	University of Manitoba, Winnipeg, Canada
Data accessibility	The raw and normalized data are available from NCBI׳s Gene Expression Ominbus (GEO) database with accession number GSE41949.

**Value of the data**

• Large scale analysis of oxidative modification of seed stored mRNAs in dormant and after-ripened (non-dormant) seeds of wheat.
• Genes corresponding to mRNAs identified as differentially oxidized by after-ripening might be involved in the control of seed dormancy.
• The data set has a paramount significance in enhancing meta-analysis and provides an important platform for comparison of similar datasets derived from seeds of other crop species.

## 1 Data

This study was performed to gain insights into the molecular mechanisms involved in the regulation of seed dormancy in wheat. Microarray based seed stored mRNA oxidation profiles of dry dormant and after-ripened (non-dormant) seeds of wheat were compared. The mRNA samples were extracted from three independent biological replicates of the two seed samples and then subjected to isolation of oxidized seed stored mRNAs. Using microarray experiments that involved the use of the Affymetrix GeneChip Wheat Genome Array and 100 ng of oxidized mRNA samples, the study presented a profile of oxidative modification of seed stored mRNAs in response to after-ripening, a period of dry storage that releases seeds from the state of dormancy. The raw and normalized files of the data are available at NCBI׳s Gene Expression Ominbus database (https://www.ncbi.nlm.nih.gov/geo/query/acc.cgi?acc=GSE41949). Detailed description of the data can be found in Gao et al. [1].

## 2 Experimental design, materials and methods

### 2.1 Plant materials and growth conditions

Wheat plants of cv. AC Domain, a hard red spring wheat cultivar with high level of dormancy [2], were grown in a greenhouse at 18–22 °C/14–18 °C (day/night) under a 16/8 h photoperiod until harvest as described previously [1]. Mature seeds were harvested and divided into two groups. One group of the seeds was stored at −80 °C to maintain dormancy while the other group was stored at room temperature for 10 months to generate after-ripened seeds.

### 2.2 Isolation of total and mRNAs, and oxidized mRNAs

Isolation of total RNA was performed from three independent biological replicates of both dormant and after-ripened wheat seed samples. RNAqueous columns (Ambion, Austin, TX) were used for extraction of the total RNA samples. After checking their purity and integrity, the total RNA samples were digested with DNase (Ambion, Austin, TX, USA) for removing any genomic DNA contaminants. The total RNA samples were subsequently used for isolating mRNAs, which was conducted using PolyATract kit (Promega, Madison, WI) following the manufacturer׳s instruction. Oxidized mRNAs were then isolated from the mRNA samples using immunostaining with 8-hydroxydeoxyguanosine (OHdG) monoclonal antibody as described previously [1], [3]. Approximately 3 μg of the mRNA from each sample was used for the isolation of oxidized mRNAs.

### 2.3 Microarray experiment

Microarray analysis was performed with 100 ng of oxidized mRNA samples derived from dormant and after-ripened seeds. After cDNA synthesis and purification, biotinylated cRNA samples were prepared using the GeneChip IVT Labelling Kit and the GeneChip Sample Cleanup Module. Quality of the labelled cRNAs was examined using an Agilent 2000 Bioanalyzer. Following fragmentation, labelled cRNA samples were hybridized for 16 h at 45 °C on GeneChip Wheat Genome Array. GeneChips were then subjected to washing and staining procedures using the Affymetrix Fluidics Station 450. Scanning of the GeneChips was performed with Affymetrix Scanner 3000.

### 2.4 Data analysis

**Fig. 1:**
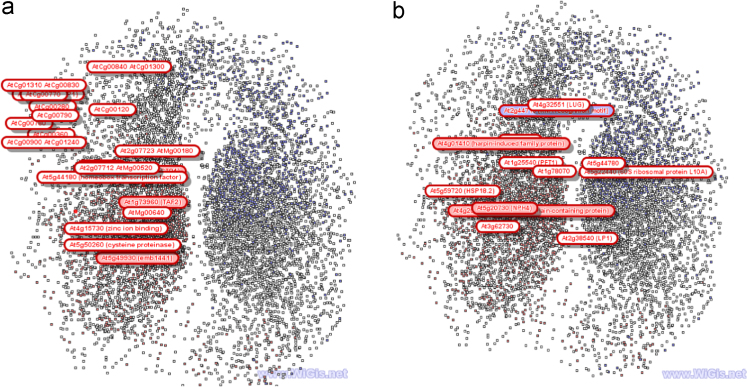
Distribution of genes highly oxidized in dry dormant (a) and after-ripened (b) wheat seeds in SeedNet topology consisting of gene sets associated with dormancy (red) and germination (purple) (http://vseed.nottingham.ac.uk; [7]).

Normalization of the raw data files was carried out with Robust Multi-Array Average (RMA), and annotation of the probesets was performed using HarvEST WheatChip (http://harvest.ucr.edu/) [4]. Identification of probesets that are differentially oxidized between dormant and after-ripened seeds was performed by analysis of variance (ANOVA) using FlexArray software [5]. Probesets were considered to be differentially oxidized if they showed ≥2-fold changes at probability level of ≤0.05. Gene ontological analysis of the probesets oxidized in response to after-ripening, which was performed using the AgriGO analysis toolkit [6], indicated their distribution over different functional categories [1]. Analysis of the Arabidopsis genes corresponding to the wheat probesets representing mRNAs differentially oxidized between dormant and after-ripened seeds using SeedNet (http://vseed.nottingham.ac.uk; [7]) revealed that genes corresponding to the oxidized probesets are overrepresented in a region consisting of gene sets associated with seed dormancy (Fig. 1).
